# Millimeter-Wave Smart Antenna Solutions for URLLC in Industry 4.0 and Beyond

**DOI:** 10.3390/s22072688

**Published:** 2022-03-31

**Authors:** Abdul Jabbar, Qammer H. Abbasi, Nadeem Anjum, Tahera Kalsoom, Naeem Ramzan, Shehzad Ahmed, Piyya Muhammad Rafi-ul-Shan, Oluyemi Peter Falade, Muhammad Ali Imran, Masood Ur Rehman

**Affiliations:** 1 James Watt School of Engineering, University of Glasgow, Glasgow G12 8QQ, UK; qammer.abbasi@glasgow.ac.uk (Q.H.A.); muhammad.imran@glasgow.ac.uk (M.A.I.); masood.urrehman@glasgow.ac.uk (M.U.R.); 2 Department of Software Engineering, Capital University of Science and Technology, Islamabad 44000, Pakistan; nadeem.anjum@cust.edu.pk; 3 School of Computing, Engineering and Physical Sciences, University of West of Scotland, Paisley G72 0LH, UK; tahera.kalsoom@uws.ac.uk (T.K.); naeem.ramzan@uws.ac.uk (N.R.); 4 School of Business and Creative Industries, University of West of Scotland, Paisley G72 0LH, UK; shehzad.ahmed@uws.ac.uk; 5 Cardiff School of Management, Cardiff Metropolitan University, Cardiff CF5 2YB, UK; 6 Tempfad Limited, London E10 6PG, UK; o.p.falade@qmul.ac.uk

**Keywords:** 5G, 60 GHz, Industry 4.0, millimeter-wave communication, smart antennas

## Abstract

Industry 4.0 is a new paradigm of digitalization and automation that demands high data rates and real-time ultra-reliable agile communication. Industrial communication at sub-6 GHz industrial, scientific, and medical (ISM) bands has some serious impediments, such as interference, spectral congestion, and limited bandwidth. These limitations hinder the high throughput and reliability requirements of modern industrial applications and mission-critical scenarios. In this paper, we critically assess the potential of the 60 GHz millimeter-wave (mmWave) ISM band as an enabler for ultra-reliable low-latency communication (URLLC) in smart manufacturing, smart factories, and mission-critical operations in Industry 4.0 and beyond. A holistic overview of 60 GHz wireless standards and key performance indicators are discussed. Then the review of 60 GHz smart antenna systems facilitating agile communication for Industry 4.0 and beyond is presented. We envisage that the use of 60 GHz communication and smart antenna systems are crucial for modern industrial communication so that URLLC in Industry 4.0 and beyond could soar to its full potential.

## 1. Introduction

The fourth industrial revolution (Industry 4.0) is a paradigm of the cyber-physical world whose philosophy is based on fully automated and digitalized smart factories for enhanced production and customized user experience [1,2,3]. Various key enabling technologies are involved in one way or the other to bring the concept of Industry 4.0 to operation, such as cloud computing [4,5], big data [6], Industrial Internet of Things (IIoT) [4,5,7], digital twins [8], artificial intelligence [9,10,11,12,13], smart communication [14,15,16,17], additive manufacturing [18,19], advanced robotics [20,21,22], and cyber-physical systems [7,8]. The main objective of Industry 4.0 revolves around automation and mass-productivity without much human intervention. On the other hand, a more human-centric approach has been envisioned to propose a new generation of the industrial revolution, i.e., Industry 5.0 [23,24,25]. Its aim is to leverage human creativity in addition to the intelligence of machines [23,24]. However, it is instructive to mention here that since the philosophy of Industry 5.0 is based on human involvement back in an industrial environment, thus on technological grounds, most of the enabling technologies that serve to visualize Industry 4.0 can equally be employed to pursue Industry 5.0. Therefore, onwards in this paper, we refer to both industrial regimes collectively as Industry 4.0 and beyond.

**Figure 1:**
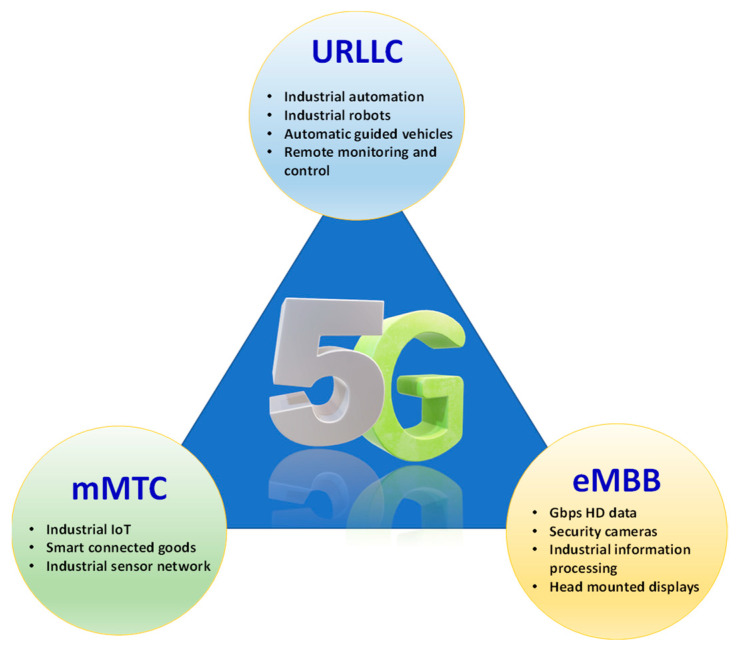
Avenues of 5G services in the viewpoint of Industry 4.0 and beyond.

In the industrial regime, the communication network has already welcomed a shift from wired to wireless communication due to the unblemished benefits of wireless infrastructure, such as mobility, scalability, easy installation, and low cost [15,26,27]. Primarily, the industrial wireless communication infrastructure is based on sub-6 GHz industrial, scientific and medical (ISM) bands, such as 2.4 and 5 GHz [28,29,30,31,32,33]. However, under the ambit of Industry 4.0 and beyond, factory automation and smart manufacturing applications require high throughput, ultrahigh reliability with a packet error rate of up to 10^–9^, low latency of below millisecond level, and agility [34]. This requirement can be linked with one of the use cases of fifth-generation (5G) mobile technology, i.e., ultra-reliable low-latency communication (URLLC), as shown in Figure 1. Out of three vertices of a 5G triangle, i.e., enhanced mobile broadband (eMBB), massive machine-type communication (mMTC), and URLLC, the factory automation scenario fits best under URLLC category to ensure millisecond-level delay in smart communication [35,36]. Moreover, with the emergence of ever more sophisticated applications (such as holographic telepresence, virtual reality, augmented reality, visual capabilities for smart robots and automatic guided vehicles, mass customization and personalization of products), the sixth generation of wireless technology (6G) has also emerged as an enabling technology to enhance the efficiency and scalability of the communication system [37,38,39,40,41,42,43,44]. As a result, traditional sub-6 GHz wireless communication cannot meet URLLC requirements of Industry 4.0 and beyond because the unlicensed spectral resources in the low-frequency bands (e.g., 2.4 GHz and 5 GHz) are limited. The existing sub-6 GHz IEEE 802.11 wireless local area network (WLAN) standards (e.g., IEEE 802.11n, IEEE 802.11ac, etc.) can only provide a restricted data rate for new emergent applications.

**Figure 2:**
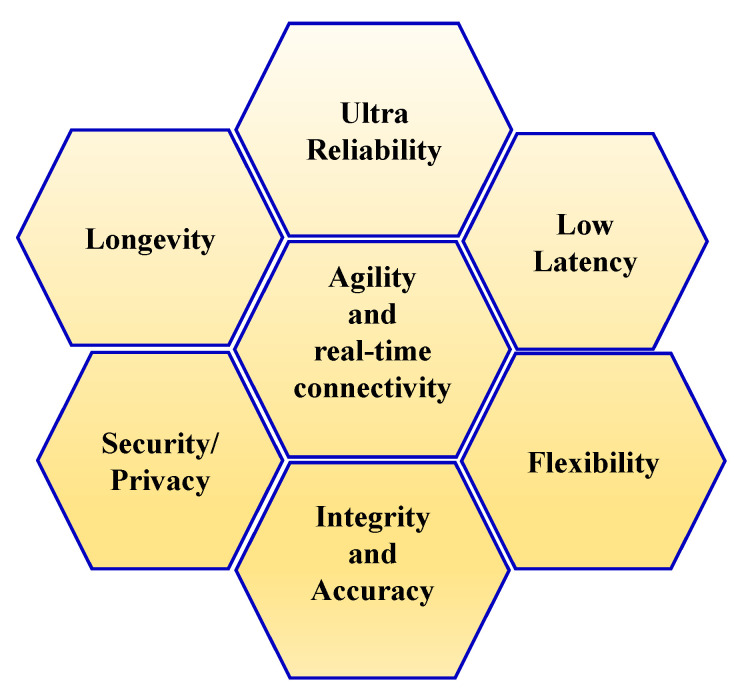
Key performance indicators for Industry 4.0 and beyond communication.

In the context of Industry 4.0 and beyond, the requirements for quality of service (QoS) and quality of data (QoD) are much more stringent [28,41]. QoS mainly includes high reliability, real-time operation, agility, seamless connectivity, security, and privacy. QoD, on the other hand, includes validity, accuracy, and integrity of the data [28]. The 60 GHz mmWave communication carries a promising potential to provide high QoS and QoD. Some of the major key performance indicators in this view are illustrated in Figure 2.

Since more spectra and new technologies are required to provide larger data rates for users to meet the ever-increasing high-speed wireless communications; therefore, industrial wireless communication is envisaged to embrace the 60 GHz millimeter-wave (mmWave) ISM band as an enabling technology to support URLLC [36,45,46,47,48,49,50].

In the mmWave regime, the 60 GHz ISM band carries a huge potential through which a wide variety of industrial automation applications can be realized. Wireless Gigabit Alliance (WiGig) is an industrial consortium that dictates the 60 GHz unlicensed band with multi-gigabits per second (mGbps)-level data rate under IEEE 802.11ad and IEEE 802.11ay standards [45,46,51,52,53]. WiGig is the next-generation Wi-Fi standard for the 60 GHz band envisioned to unleash URLLC in the indoor factory environment. It offers a huge bandwidth of 14 GHz, which is way more than all sub-6 GHz unlicensed bands combined. Moreover, the standardization of the new radio access technology (RAT) for 5G systems, called New Radio (NR) under the Third-Generation Partnership Project (3GPP), supports mmWave carrier frequencies. In this context, the considerations to allow NR to access unlicensed spectrum (NR-U) of 60 GHz are anticipated [48,54,55]. Details about the waveforms and numerologies of 5G NR are discussed in detail in [56,57].

A number of surveys, tutorials, and reviews are presented in the literature about various technologies associated with Industry 4.0 and beyond [1,4,6,7], mmWave communication [45,46,51,52,53,58,59,60,61,62], and mmWave antenna designs [63,64,65]. Discussions about IoT, cyber-physical systems, big data, health care for Industry 4.0, and revolutionary phases toward Industry 5.0 are presented in [1,4,6,7]. A survey of enabling technologies in Industry 5.0, such as edge computing, digital twins, collaborative robotics, Internet of Everything, blockchain, and 6G and beyond networks, is presented in [24]. For 60 GHz transceiver design, advances in the design of mmWave circuit components such as power amplifiers, low-noise amplifiers, analog-to-digital converters, on-chip, and in-package antennas are discussed in [59]. A survey of solutions and standards used in designing architectures and protocols for mmWave communications is reported in [61]. A review of multibeam antennas with beamforming systems is presented in [64]. A review of mmWave beamforming and MIMO is presented in [45,60,61,62]. The wireless protocols and standards (such as IEEE 802.11ad/ay) working at 60 GHz are reviewed in [45,51,52,53]. An inclusive review of mmWave array antennas for various mmWave applications is presented in [63,65] but without the demands and context of Industry 4.0 and beyond. However, it is noticed that a combined study of the requirements of Industry 4.0 and beyond, potential frequency bands, and related smart antenna solutions for them is missing in the literature. Consequently, to address this gap, here we first present the review of industrial communication in the context of Industry 4.0 and beyond, then highlight the potential of the 60 GHz mmWave band for future industrial communications, and finally review various smart antenna designs at 60 GHz for Industry 4.0 and beyond communication. A summary of the earlier contributions to Industry 4.0 and beyond, mmWave communication, and mmWave antenna designs are depicted in Table 1.

### 1.1. Contributions

In this review paper, first, we discuss the impediments of sub-6 GHz communication from the viewpoint of Industry 4.0 and beyond. We review and highlight the potential prospects of 60 GHz mmWave communication holding up URLLC in Industry 4.0 and beyond applications. We discussed stringent communication requirements and fundamental key performance indicators of Industry 4.0 and beyond. The advantages and challenges of a 60 GHz unlicensed ISM band for industrial communication are reviewed in detail. Then we discussed standards and protocols operating at the 60 GHz band suitable for antenna designing. After establishing the communication requirements and frequency bands, we then review the state of the art of smart antennas for 60 GHz mmWave communication in the industrial environment. In the end, we highlight some research opportunities and challenges related to mmWave communication in the industrial environment. The contributions of this review paper are summarized as follows:

- We present an overview of the sophisticated applications under the ambit of Industry 4.0 and beyond;
- We reveal various limitations of sub-6 GHz ISM bands that can not meet the stringent requirements of modern industrial applications;
- We explore various key performance indicators (KPI) to ensure URLLC in Industry 4.0 and beyond. Based on these KPIs, we highlight the potential of the 60 GHz mmWave ISM band based on state-of-the-art literature;
- We investigate the potential advantages as well as the challenges of 60 GHz mmWave communication;
- We identify different standards and protocols working at unlicensed 60 GHz ISM bands so that smart antennas for Industry 4.0 and beyond can be designed at these bands. This might help antenna design engineers to select the right frequency bands to target 60 GHz mmWave industrial communication;
- By establishing the potential of the 60 GHz mmWave band for smart industrial communication and highlighting the wireless standards, we review various 60 GHz mmWave antenna designs and discuss their challenges for Industry 4.0 and beyond applications;
- We emphasize the intriguing potential emerging in this domain, explaining new design characteristics and research paths for the researchers in this domain. As a result, this review paper can act as a catalyst for more research into 60 GHz mmWave smart antenna designs and the development of physical layer-based solutions to support smart communication in the era of Industry 4.0 and beyond.

### 1.2. Paper Organization

**Figure 3:**
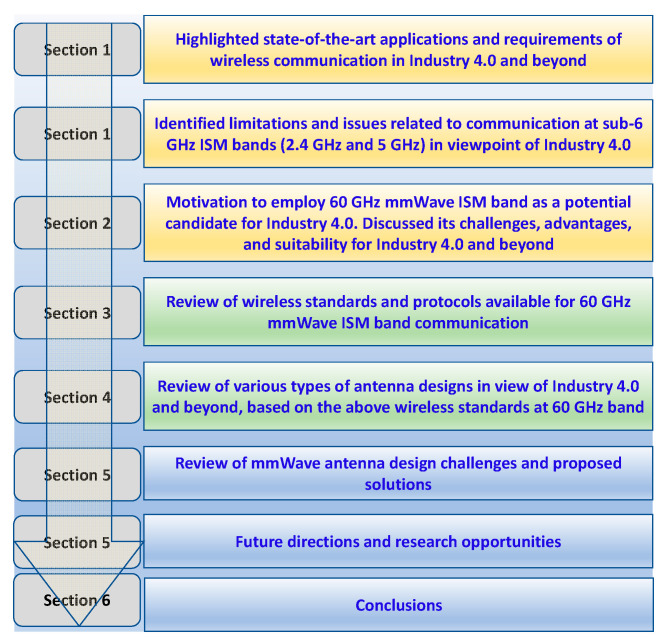
Organization of this review paper.

The rest of the paper is organized as follows: Section 2 presents the potential prospects, advantages, and challenges in 60 GHz band communication in an industrial environment. Section 3 discusses standards and protocols for the 60 GHz mmWave ISM band for smart industries. Section 4 reveals state-of-the-art antenna designs, their challenges, and design considerations in the 60 GHz band. Section 5 unveils research prospects and future directions. Section 6 concludes the paper. The organization of this paper is depicted in Figure 3.

## 2. Advantages and Challenges of mmWave Industry 4.0 and beyond Communication

### 2.1. Advantages of mmWave Communication

#### 2.1.1. Large Available Bandwidth

The large available bandwidth of 60 GHz unlicensed band unfolds many applications for Industry 4.0 and beyond. Some of the sophisticated smart industrial applications include vision-guided robots, ultrahigh definition video and imaging for remote visual monitoring, smart safety instrumented systems, intelligent logistics, and high precision image-guided automated assembly, to name a few [45,46]. The capacity to achieve URLLC in factory automation scenarios allows smart robots and machines to work alongside humans or collaborate on a similar goal. However, with the potential use of mmWave high throughput communication, a seamless adaptive behavior can be employed in the machines/equipment to make them capable of detecting adjacent individuals or objects and reacting appropriately, such as by modifying their movements, slowing their operating rate, or even halt completely.

#### 2.1.2. Inherent Security

The 60 GHz communication is sensitive to blockage due to weak diffraction capability around the objects in an industrial environment [67]. Moreover, because of beamforming, the nature of communication is highly directional. Even a small misalignment between the receiver and transmitter can lead to communication deafness. However, this aspect can be positively used as a security enabler. This also helps in avoiding jamming signals due to the directionality of the transmitter and receiver antenna systems. The problem of jamming detection and mitigation in 5G-enabled Industry 4.0 indoor factory deployments is presented in [68].

#### 2.1.3. Efficient Spectrum Reuse

Since mmWave beamforming with large arrays produces highly directional beams, thus the effect of co-channel interference is greatly reduced. To benefit from this inherent feature, MIMO or multibeam antenna techniques can be employed to reuse the spectrum resource and produce redundance radiation beams. This will provide diversity gain and multiplexing gain, in addition to array gain [69]. This way, spatial redundancy, and diversity can be efficiently used to reuse the spectrum in industrial communication.

#### 2.1.4. Beamforming

Since 60 GHz communication is directional, thus beamforming is needed to obtain narrow beams. In antenna beamforming, an array of multiple antenna elements is used to produce a net-directed beam. One of the most significant advantages of mmWaves is that directional transmission greatly reduces the interference problems that hinder the sub-6-GHz bands, resulting in increased throughput. Moreover, co-channel interference is not a problem in mmWave communication; thus, mmWave networks primarily function in noise-limited rather than interference-limited mode, which makes it easier for collaborative robots to communicate at the same time in the smart industrial environment.

#### 2.1.5. Size Miniaturization

**Figure 4:**
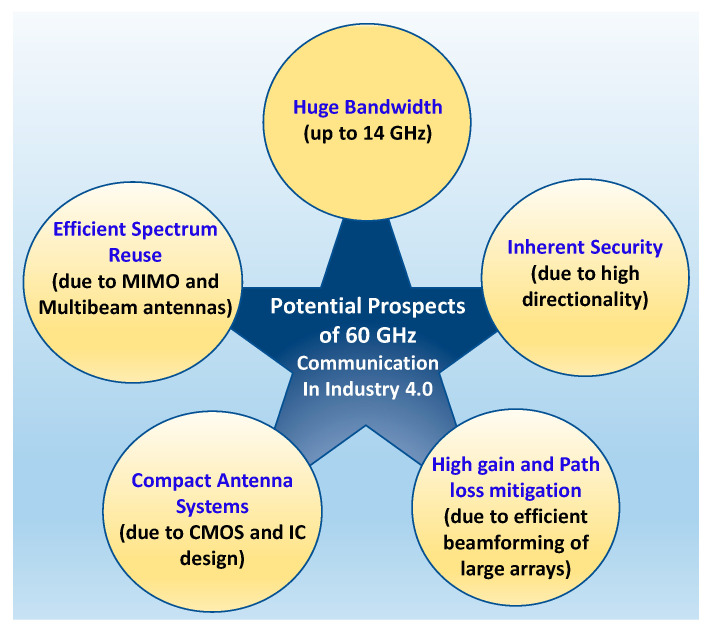
Advantages of 60 GHz mmWave communication in Industry 4.0 and beyond.

The advancement in complementary metal oxide semiconductor (CMOS) technology and integrated circuits (IC) designs in recent decades have paved the way to realize compact 60 GHz transceivers and radio designs [59]. Since the Federal Communication Commission (FCC) and other governing organizations released the 60-GHz spectrum, great progress has been made toward the realization of highly integrated 60-GHz radios for low-cost, potentially ubiquitous consumer adoption. Some comprehensive surveys on 60 GHz radios, transceivers, on-chip antennas, and other related circuitry are presented in [59,66,70]. The advantages of 60 GHz mmWave communication are summarized in Figure 4.

### 2.2. Challenges of mmWave Communication

#### 2.2.1. LOS Blockage

Industrial wireless communication is different from that of commercial, home, or office wireless communication. This is due to the large metallic structures, heavy machinery, large pillar structures, and random moving objects. Thus, an industrial environment is usually considered as a heavy multi-path and rich scattering [45]. In this view, the narrow beam antennas with beamforming reduce the available path diversity and weaken non-line-of-sight (NLOS) scenarios, which may sometimes cause blockage. Moving robots, human activity, or any other large scatterers and absorbers result in a fast-fading channel. Thus, characterizing radio propagation behavior in realistic factory environments is difficult due to shorter mmWave wavelengths, which cause channel properties to be sensitive to the actual topology and size of surrounding objects. As a result, accurate channel models are required to be used for accurate communication systems [49,50,71,72,73].

#### 2.2.2. Path Loss

mmWave propagation at 60 GHz faces severe path loss because of oxygen absorption, as well as according to Friis’ equation. The free-space propagation loss is directly proportional to the square of the frequency. The path loss at 60 GHz is about 21 to 28 dB (at 10 m distance) more as compared to conventional sub-6 GHz communication bands [45,52]. Furthermore, 60 GHz communication exhibits quasi-optical propagation, in which the received signal is dominated by the LOS path and first-order reflections from strong reflecting materials [74].

In order to combat path loss issues, 60 GHz communication leverages the beamforming antenna gain. As a result, array gain helps to improve the signal-to-noise ratio (SNR) to mitigate path loss at 60 GHz. Thanks to the smaller wavelength at 60 GHz, array form factors are much lower as compared to those of sub-6 GHz designs. Moreover, for the same physical antenna size, mmWave transmissions allow for a better antenna gain to mitigate path loss [69]. An improvement of 28.1 dB in path loss was achieved using a multibeam antenna system by combing the strongest received signals coherently at mmWave bands [75]. Besides the antenna array gain, the implementation of multiple-input-multiple-output (MIMO) in IEEE 802.11ay also helps to enhance the multiplexing gain. However, due to directional communication using beamforming, interference can occur if the receive beam aligns with unwanted transmitters, causing potential disruption.

## 3. Wireless Standards at 60 GHz mmWave Band

**Figure 5:**
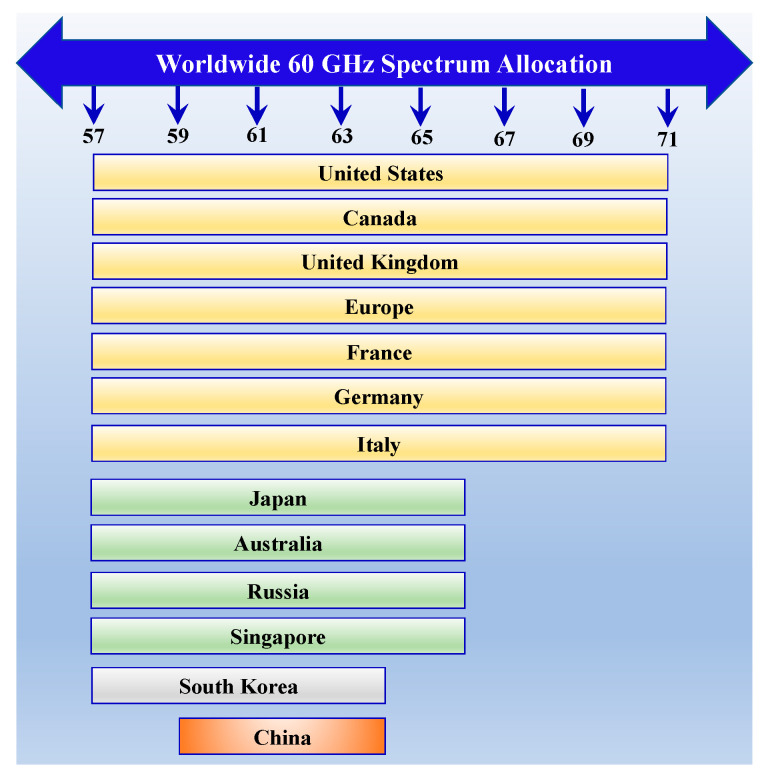
Worldwide allocation of 60 GHz mmWave spectrum.

Different standardization efforts are being made for 60 GHz communication. Current standards include IEEE 802.11ad, IEEE 802.11ay, IEEE 802.15.3c, WirelessHD and ECMA387 [51,59,76,77]. However, ECMA, WirelessHD, and IEEE 802.15.3c are wireless personal area network (WPAN) standards. The two most anticipated WLAN technologies for ultrahigh-speed 60 GHz communications are IEEE 802.11ad and IEEE 802.11ay. These are extended protocols of the IEEE 802.11 Wireless Fidelity (Wi-Fi) family. As opposed to the limited available bandwidth of sub-6 GHz ISM bands, such as 2.4 GHz and 5 GHz with 85 MHz and 500 MHz of bandwidth, respectively, the 60 GHz mmWave ISM band provides huge bandwidth of up to 8 GHz. IEEE 802.11ad and IEEE 802.11ay are networking standards for WiGig communication networks for supporting data rate of mGbps, which are anticipated as key enablers for indoor factory automation communication as well. However, 60 GHz communication is supposed to be directional, supported by beamforming techniques as opposed to sub-6 GHz omnidirectional communication. The complete 60 GHz band covers the frequency range from 57 to 71 GHz, according to a new release by FCC [78]. There has been a release of 14 GHz spectrum in the United States, Europe, and Canada. However, the 9 GHz spectrum (57–66 GHz) is being used in other regions such as Singapore, Australia, and Russia. China uses a 59–64 GHz band, whereas 57–64 GHz is being used in South Korea. A worldwide spectrum allocation of the 60 GHz unlicensed band is depicted in Figure 5 [78,79]. A summary of 60 GHz wireless standards is given in Table 2 [59].

### 3.1. IEEE 802.11ad

IEEE 802.11ad is a standalone unlicensed protocol that provides data rates up to 8 Gbps. It spans from 57 to 71 GHz with six sub-divided channels, each with a bandwidth of 2.16 GHz. It supports only single-channel transmission of 2.16 GHz bandwidth and does not support multi-user MIMO (MU-MIMO). It uses carrier sense multiple access with collision avoidance (CSMA-CA) [77]. There are different modulation and coding schemes that can be grouped together for high SNR, power efficiency, low complexity, robustness, and high achievable data rates. The idea of antenna sectors is introduced in 802.11ad, which corresponds to a discretization of the antenna space, reducing the number of possible beam directions to test. This is analogous to beamforming. The sector-level sweep (SLS) phase and the beam refinement protocol (BRP) phase are the two steps of beamforming training. The SLS is required to establish a link between the two stations, with one station attempting different antenna sector configurations consecutively while the other has its antennas set up in a quasi-omnidirectional fashion. In the case of phased antenna arrays, the BRP is then used to fine-tune this arrangement by employing narrower beams and possibly optimizing the antenna weight vectors.

For a pair of transmitter-receiver stations, beamforming training identifies the optimum receive and transmit antenna sectors. A bidirectional training frame sequence is transmitted to accomplish this. IEEE 802.11ad may achieve a maximum throughput of up to 8 Gbps with a fully trained transmit and receive antenna beamforming configuration. Furthermore, the beamforming protocol provides a low antenna gain device training procedure and can send training parameters to a central network coordinator for channel access scheduling. A detailed analysis of this protocol has been presented in [52].

### 3.2. IEEE 802.11ay

IEEE 802.11ay is an amendment to IEEE 802.11ad standard, which supports faster and longer-range mGbps communication. The hallmark of this standard is its ultrahigh performance for fixed point-to-multipoint (P2MP) and point-to-point (P2P) transmissions, either indoor or outdoor. It is a standalone unlicensed protocol that provides a theoretical peak data rate of up to 100 Gbps. In addition, it provides a bandwidth of 8.64 GHz as well as supports MU-MIMO. It supports channel bonding and channel aggregation [51,58] to provide higher throughput and bandwidth. Channel bonding corresponds to when many contiguous channels are merged into a single wideband channel, there is no channel spacing between them, and they can be used as a whole hand to form a single channel. Channel aggregation, on the other hand, is frequently used to combine two or more contiguous or non-contiguous channels with channel separation between them.

Additionally, in IEEE 802.11ad, the MIMO link can accommodate up to eight spatial streams per station, depending on the environment, the antenna’s directivity, and whether or not antenna polarization is used. Multiplexing gain is added to the beamforming gain by employing antenna arrays in MIMO communication. As a result, the spatial domain’s variety is completely exploited, enhancing communication durability and resulting in extremely high SNR links that are almost resistant to fading. A detailed analysis of beamforming training, MAC schemes, channel bonding, and radio propagation of IEEE 802.11ay is described in [51].

## 4. Antennas for mmWave Industry 4.0 and beyond Communication

Antenna design at the 60 GHz mmWave band has been explored to a great extent in recent years. This is due to the advancement in CMOS and IC design technologies that support compact mmWave circuitry and RF frontends. Array antenna systems with beamforming are essential to support robust and reliable communication due to smaller wavelengths at mmWave and high susceptibility to path loss. The beamforming antennas help to improve the coverage, SNR, and effective beam steering [80]. We broadly categorize the 60 GHz smart beamforming antennas into three types: printed circuit board (PCB)-based antennas [80,81,82,83,84,85,86,87,88,89,90,91,92,93,94,95,96,97], on-chip antennas [59,98,99,100,101,102,103], and low temperature co-fired ceramic (LTCC)-based antennas [104,105,106,107,108,109,110,111,112,113,114,115]. We discuss these antennas here in detail.

### 4.1. PCB-Based Antennas

PCB technology is cost-effective and easy to fabricate for antenna designs [65]. For mmWave antenna design, ease of fabrication and integration is of paramount importance. PCB technology is a common choice to fulfill these requirements. Moreover, the radiation properties of a particular antenna element are also an important consideration in the technology selection process. Broadside antennas are typically easier to integrate into array configurations, whereas end-fire radiating elements lend themselves best to edge integration. Microstrip patch antennas are currently among the most used radiating elements for 60 GHz mmWave arrays, despite material losses and performance limitations at this band. Various substrates are used to design such antennas, such as FR4, Rogers, liquid crystal polymers (LCP), polytetrafluoroethylene (PTFE), etc. [81,86,116,117,118]. For industrial communication, some other durable substrates are also reported in the literature, such as Arlon, but at lower frequency bands [119]. However, conductor and dielectric losses are quite high in PCB-based antennas at mmWave frequencies.

Because of the skin effect at 60 GHz, conductor losses increase manifold. A survey on various mmWave antenna designs from 10 to 100 GHz is presented in [65]. Many conventional PCB-based antennas such as microstrip patch, dipole, monopole and slot antennas are reported in the literature [81,85,86,92,95,97,120,121]. Moreover, Vivaldi antennas [122,123,124], dielectric resonator antennas [120,125,126], fractal antennas [127,128,129,130], and substrate-integrated waveguide (SIW)-based leaky wave antennas [96,127,131,132,133] are also implemented on PCB technology.

The key to enhancing antenna gain at the mmWave band is to use array configuration. A detailed overview of various mmWave array antennas, different array geometries, and the challenges in array configuration is presented in [65,134]. Microstrip-based array antennas at 60 GHz may suffer losses. The properties of the substrate and its thickness necessitate extra consideration. At 60 GHz, the substrate thickness is quite high when compared to sub-6 GHz microwave frequencies. This impacts the bandwidth and input impedance of the antenna. Moreover, proper feed structure is essential to be designed for accurate antenna performance [65]. Furthermore, a review of array antennas, including 60 GHz P2P and P2MP communication, is provided in [92].

### 4.2. LTCC-Based Antennas

LTCC technology has emerged in recent years for the miniaturization of portable electronics devices. It is a multilayer technology and eludes many disadvantages of PCB-based designs. LTCC is a multilayer technology that has been used for packaging ICs and has been applied to sensors, actuators, and integrated microsystems with relatively low cost and high productivity [106,109,135]. LTCC-based antennas can be employed with efficient characteristics in industrial environments as well. Various circularly polarized (CP) and linearly polarized (LP) LTCC-based antennas are reported [104,107,110,111,112]. Since the electric field of the wave only changes direction in a circular fashion in circularly polarized antennas, the signal strength remains constant. Thus, high gain CP antennas can be more useful for effective short-range communication in a rich scattering industrial environment. An LTCC-based phased array antenna at 60 GHz integrated with a 28 nm CMOS transceiver at 60 GHz was reported in [136].

In order to mitigate the high cost of large arrays at the 60 GHz band for beamforming or MIMO, a metamaterial-based LTCC compressed Luneburg lens antenna is reported at the 60 GHz band [113]. An assembly of stacked multilayer PCB was used to realize a high gain LTCC-based Luneburg lens antenna. A comprehensive review of various LTCC-based antenna designs at 60 GHz is presented in [106]. In the future, LTTC-based antenna designs can be employed in Industry 4.0 and beyond at 60 GHz as a low-cost and compact solution.

### 4.3. On-Chip Antennas

As a result of unprecedented development in integrated circuit (IC) design due to the unmatched level of integration and aggressive scaling of transistors in the last few decades, on-chip antennas have become part of the chip. Earlier, the system-in-package (SiP) approach was mainly used to include antenna design along with the chip. However, with this approach, the antennas, which have diameters on the order of wavelengths, are still outside the package and are still the bottleneck for true on-chip system realization [102]. More advancements and improvements in complementary metal oxide semiconductor (CMOS) and silicon germanium (SiGe) technologies have paved the way for the design of small-scale packaged antennas on silicon wafers using the system-on-chip (SoC) approach [59,102]. Moreover, for PCB-based antennas, the bond wires are used to connect antennas to ICs because they are typically implemented on PCBs. As a result, because these bond wires are not well characterized, matching can suffer greatly, especially at higher frequencies GHz band. In this way, on-chip antennas helped to alleviate this problem [102].

On-chip antenna configuration is governed by foundry-specific constraints, whereas on-chip antenna characterization necessitates the use of particular text fixtures. In recent years, the majority of on-chip antennas have been implemented in bulk silicon-based technologies such as CMOS and SiGe (with low resistivity of 10 ohm-cm), as opposed to other semiconductor technologies (with high resistivity) such as gallium arsenide (GaAa) [59,102].

Owing to smart manufacturing and integration techniques, these on-chip antennas can be integrated with moving objects, smart robots, moving machines, unreachable points in a factory scenario, and/or human head-mounts to provide indoor seamless connectivity at 60 GHz. Various on-chip antennas have been reported in the literature [59,101,102], among which monopole, dipole, loop, and Yagi-Uda antennas are fundamental types. An insightful demonstration of on-chip antennas, their design rules, characterization, limitations, challenges, and solutions are presented in [102]. The 60 GHz on-chip antennas, as well as the complete RF front end, are reviewed in [59]. The advantages and disadvantages of different types of 60 GHz mmWave antennas are summarized in Table 3. A comparison of various 60 GHz antennas is given in Table 4.

## 5. Research Opportunities and Future Directions

Where there are challenges, there are opportunities. Millimeter-wave communication has opened many research opportunities in wide areas of communication systems. Some key opportunities and open research questions are discussed in this section.

### 5.1. RF Frontend Design

First, there exists a huge room for improvement and advancements to design efficient RF frontend components. For instance, mmWave power amplifiers are usually less efficient due to the very high frequency of operation. Similarly, the design of wideband PAs and low-noise amplifiers (LNAs) at 60 GHz mmWave is also a challenge [137]. Particularly high PA efficiency will help to extend communication range and enhance battery life. The phase shifters (e.g., to be used for phased array antennas) at mmWave frequencies are quite lossy, and thus, accurate phase shifts are difficult to achieve with low insertion loss. Lossy phase shifters also reduce antenna gain at high frequencies. Thus, research should be carried out to design efficient phase shifters at mmWave bands to reduce antenna losses. In the same way, other RF components, such as mixers and voltage-controlled oscillators, must have a very large tuning range at the 60 GHz band. Likewise, the design of high-speed digital to analog converters (DAC) and analog-to-digital converts (ADC) is also a challenge. ADC is a crucial component of the 60 GHz communication system as it determines the achievable data rates.

### 5.2. System-on-Chip Design

The advancement in CMOS, SiGe, and IC design has led to the design of on-chip antennas (AoC) and antennas-in-package (AiP). AoC has always remained a bottleneck for the realization of true system-on-chip (SoC) RF solutions. Efficiency is the main issue in AoC due to the low resistivity and high permittivity of a silicon substrate [102]. However, some solutions, such as CMOS-MEMS (microelectromechanical systems), to suspend the antenna structure in air and keep a gap from the silicon substrate, may increase the efficiency and gain of AoC [98,102]. Moreover, the characterization and measurements of AoC are also different than those of conventional PCB-based antennas. This is because on-chip antennas are fed using small wafer probes, which are fragile and prone to damage. Any mishandling may affect the measurement results. A microscope is required to accurately place the probes on the miniature on-chip antennas or the lines feeding the antenna. Microscopes are not standard anechoic chamber equipment, so they must be carefully placed so that they do not interfere with the antenna radiation or obstruct its movement [102].

### 5.3. mmWave Antenna Array Design Challenges

At mmWave frequencies, array antennas are crucial for high gain and directional communication. Array designs at mmWave frequencies face additional challenges compared to low-frequency arrays [138]. The antenna element is an important part of the phased array antenna design. It has to be chosen according to the requirements of the antenna array, such as operating bandwidth, gain, sidelobe levels (SLL), polarization, etc. [139]. Extra emphasis needs to be placed on array antenna gain (to compensate for high attenuation and path loss of mmWave), size, and interoperability with other radio frequency modules in mmWave communication systems [65,139]. The high directivity of mmWave beams should ensure that interference is minimized. The substrate materials used in mmWave array antennas also affect their performance. The thermal capacity of the substrate material also limits the power. Radiation pattern, bandwidth, input impedance, and efficiency are all influenced by conductor characteristics, substrate thickness, and relative permittivity values of the substrate [65,134].

Due to closely placed antenna elements in array configuration, the mutual coupling in proximity becomes an issue for antenna design engineers and needs special attention [65]. Mutual coupling between array elements causes grating lobes and power transfer from one element to another via direct radiation and surface waves. Careful selection of the gap between the antenna elements is necessary to avoid grating lobes [140]. In some cases, grating lobes are deliberately introduced to combat the sparsity of mmWave channels with an analog beamformer [141]. These intentionally created grating lobes are employed in an analog beamformer to increase the scattering intensity in NLOS settings and therefore increase the scattering in the received signal power delay profile. Mutual coupling at mmWave frequencies rises as the substrate becomes thicker, resulting in a greater number of surface wave modes. As a result, the array sidelobe levels and main beam shape are degraded and may also cause scan blindness [92]. Various methods to reduce the mutual coupling between array elements are reported in [142,143,144,145,146,147].

The polarization of the array antenna is also an important factor. Polarization is defined by the time-varying electric field vector at a given observation point. To overcome the polarization mismatch, multi-polarized antenna arrays are used with the improvement in diversity gain [148,149,150]. Polarization is also affected by mutual coupling. As the scan angle increases, the mutual coupling causes cross-polarization isolation to deteriorate [151]. To maintain polarization purity, polarization compensation techniques are sometimes used, which increase the cost and complexity of mmWave array design [152,153,154,155].

Appropriate feeding mechanisms and power division networks should be designed, and their effects should be included in the antenna design process. The performance of an array is degraded by the radiation from the feed network. Mitigating the feed connector losses in mmWave PCB-based antennas is a challenging task and demands special attention. Particularly, in an industrial environment, electromagnetic interference from other machines and electrical appliances may affect the antenna performance severely. In this way, complete electromagnetic modeling of the antenna system with respect to the industrial ambiance is necessary to achieve accurate performance of the antennas. Extra care should be given to the selection of antenna element type and material properties for industrial communication.

The development challenges in mmWave antenna beamforming networks are presented in [156] that need to be addressed in future research. The review of mmWave penetration, coverage, security, scalability, attenuation, and antenna array architecture is presented in [157,158]. In order to make a robust and efficient smart antenna system, the smart antenna systems should entail rigorous RF measurements in real industrial environments. This will help to fully characterize the propagation channels of industrial wireless systems over the mmWave band.

### 5.4. mmWave MIMO

Since 60 GHz IEEE 802.11ay standard supports MIMO configuration [58]; thus, more advanced MIMO techniques should be designed and experimentally analyzed for different antenna directivities and single-polarized or dual-polarized transmissions. Moreover, suitable research opportunities exist for the design and implementation of dynamic adaptive dual-polarized antenna arrays for millimeter-wave, as well as low-power and low-complexity MIMO systems [159,160] that allow for flexible analog and digital beamforming and advanced signal processing techniques. MIMO, massive MIMO, and multiple antenna techniques at mmWave bands are hot cake topics for researchers [60].

Furthermore, for the wideband properties of 60 GHz channels, severe hurdles for MIMO communications may be created, particularly in multi-user scenarios. A detailed survey of MIMO channel models in wireless communication is presented in [161]. It is worthwhile to investigate adaptable systems that adjust to changing channel circumstances to attain the required performance. Additional beamforming gain might be obtained by using a denser array. Directivity and spatial multiplexing gain are provided by a mmWave MIMO architecture with a series of sub-arrays [162]. As a result, the diversity in the spatial domain is fully used, enhancing communication resilience and resulting in exceptionally high SNR channel links that are practically fading-proof in an industrial environment. Furthermore, MIMO with beamforming has been reported to enhance PHY security at 60 GHz [163], which is an active area of research for Industry 4.0 and beyond.

### 5.5. Beamforming

Beamforming techniques created specifically for NLOS and LOS radio channels will be required for next-generation wireless communication systems. As the size of antenna elements is much reduced at mmWave bands and a large number of antenna elements are involved in an array configuration, thus mmWave beamforming requires more RF components and RF chains, which increases the complexity of the antenna system [164]. In this view, the huge room is available to perform research to devise efficient, less complex, and low-cost analog/digital hybrid architectures for smart antenna systems. In a factory environment, multiple antenna systems with numerous radiating elements at the access point are particularly interesting options for obtaining very high data rates (mGbps) for users sharing the same spectrum at the same time [156].

Furthermore, for an effective hardware implementation of a beamformer system operating in the mmWave band, a realistic understanding of hardware impairments, wave propagation, and antenna characteristics is required in industrial communication. Contemporary research challenges in mmWave beamforming include solving complicated challenges linked to analog/digital hybrid beamforming, polarization diversity, optimization of the beam search process, concurrent beamforming protocols, resilient adaptive beamforming, exploiting channel sparsity, and 3D beamforming. These systems must achieve the goals of lowering beamforming calculation costs, delays, and power consumption while maintaining acceptable QoS to provide mGbps throughput [62].

### 5.6. Terahertz Communication

Terahertz (THz) communication is considered an enabling technology to envisage beyond-5G and 6G systems [165,166,167]. Industry 4.0 and beyond can also benefit from THz communication for future mission-critical applications with the high demand for massive URLLC [168,169,170,171,172]. However, more difficult propagation conditions exist at THz frequencies, such as increased path loss, higher penetration loss, and more severe shadowing. Besides other challenges such as standardization, design of THz sources, THz integrated circuits, and advanced THz antenna fabrication techniques [173,174,175,176].

THz antennas are the key devices for THz communication because of their tiny size, wide frequency bandwidth, and high data rate [177,178]. A plethora of THz antenna designs are reported in the literature for THz communication [177,179,180,181,182,183,184,185,186,187], to list but a few. Graphene is extensively used in THz antennas due to its exceptional optical properties [181,188]. THz communication is believed to be an emerging and enabling technology in future Industry 4.0 and beyond to provide extremely high data rates, with open research challenges [168].

### 5.7. Distributed Antenna System

The relatively harsh environment in industries (as compared to homes, offices, etc.) demands robust communication solutions. Traditional co-located multi-antenna systems reduce path loss, but they are insufficient in demanding real-world industrial applications where NLOS conditions occur often [37,189,190]. URLLC in Industry 4.0 and beyond for cyber-physical systems is a crucial challenge that is anticipated to be served by distributed antenna systems at mmWave bands [191,192,193,194]. The potential of mmWave-over-fiber distributed antenna systems for high-data wireless communication was demonstrated in [191]. Measurements in a controlled anechoic chamber and a realistic environment resembling an Industry 4.0 setting revealed the potential of distributed antenna system for establishing and maintaining mGbps wireless communication while overcoming self-blocking and LOS blockage issues [191,195].

The link blockage problem must be carefully addressed in order to transmit mmWave signals through LOS pathways to the required users. This will result in effective coverage and smooth network connectivity for mmWave communication systems [139]. Distributed antenna systems can serve as a potential solution by adding the properties of mmWave communication systems [192,196].

### 5.8. Machine Learning for Antennas in Industry 4.0 and Beyond

The potential of exploiting machine learning (ML) is imminent in 5G and beyond-enabled Industry 4.0 and beyond. At each abstraction layer, ML is anticipated to be used to predict service demands of Industry 4.0 and beyond as well as the evolution of the wireless channel in order to design self-optimizing and self-updating networks [197,198,199,200]. It is unveiled in [191] that the traditional multi-antenna signal processing approaches are no longer sufficient for broadband mmWave massive MIMO systems due to a large number of radiating elements involved, high data rates, and a high number of mobile users. Unlike traditional communication systems, which demand many computing resources and result in unacceptable latency, deep-learning-based approaches take advantage of natural channel sparsity to efficiently precode and modulate data into several streams and send it to the distributed system [191,201,202,203]. Moreover, a physics-inspired neural network is used in [204] to design a reconfigurable coded metasurface for dynamic beam steering. Such type of ML-based reconfigurable antennas possess huge potential for Industry 4.0 and beyond applications, where the smart antennas system is required to reconfigure its radiation pattern in real-time based on blockage, jamming, and NLOS scenarios.

### 5.9. Reconfigurable Intelligent Surfaces

Reconfigurable intelligent surface (RIS) is a groundbreaking technique for reconfiguring the wireless propagation environment through software-controlled reflections to achieve enhanced spectral and energy efficiency in a cost-effective manner [205,206,207,208,209,210]. Some alternative terms are also used for RIS, such as intelligent reflecting surface (IRS) or software-controlled metasurfaces [211,212]. By changing the phase and/or amplitude, the elements of RIS can separately reflect the incident signal. In this way, beamforming for directional signal augmentation or null placement is performed. Radiating elements, control circuitry using PIN diodes or varactors, and a controller or field-programmable gate array (FPGA) [213,214]. RIS technology is still in its infancy; however, most of the development aspects of RIS are presented in detail in [215,216]. Furthermore, beamforming development with RIS is studied in [217,218,219].

RIS provides a unique and cost-effective method for proactively combating wireless channel impairments. In this view, RIS-aided industrial communication is an emerging research area with huge potential [208,220]. RIS-aided wireless communication carries the promising potential to be an enabling technology to assist smart communication in Industry 4.0 and Industry 5.0 [25]. Due to densely distributed industrial equipment (such as metal machinery, unpredictable movement of things (robots and trucks), wooden structures, and thick pillars), wireless signals are easily blocked and reduce performance reliability. When the direct communication link is impeded, RIS is a viable choice to provide an alternate transmission link [21,25,221,222]. The communication link can be created using a RIS mounted on the factory ceiling or the wall, delivering mission-critical industrial communication with seamless, reliable connectivity. A vision of RIS-enabled communication in Industry 4.0 and beyond seems to become a reality in the near future.

## 6. Conclusions

The increasing demand for URLLC as a result of burgeoning sophisticated and mission-critical applications in Industry 4.0 and beyond necessitates more spectrum and new technologies. The mmWave communications appear to be the key to delivering the solution. As a result, future 5G networks and WLANs are paying close attention to mmWave communications to enable the full potential of Industry 4.0. First, we presented an overview of Industry 4.0 and the stringent communication requirements. We then highlighted the issues associated with existing sub-6 GHz industrial wireless standards and their limitations in the industrial context.

Unfurling the challenges of communication at sub-6 GHz ISM bands, we then unfolded the prospects of mmWave communication in an industrial setting. We highlighted the potential advantages, prospects, and challenges of the 60 GHz mmWave ISM band for URLLC in factory automation from the viewpoint of Industry 4.0 and beyond. The WiGig consortium has been working to widespread 60 GHz unlicensed spectrum worldwide, which can be leveraged for smart industrial communication and operations. A detailed discussion on IEEE 802.11 ad and 802.11 ay standards for multi-gigabits-per-second data rate communication is also presented. We also identified the prospects of the 60 GHz spectrum for mission-critical and low-latency applications in smart factory automation. Moreover, the propagation challenges at the 60 GHz band were also elucidated. Furthermore, it is expounded that beamforming is a crucial requirement at 60 GHz communication to ensure directional communication. In this view, smart beamforming array antennas as PHY candidates are of paramount importance. We present the review of various state-of-the-art 60 GHz mmWave antenna antennas suitable for Industry 4.0 and beyond. Moreover, several challenges of mmWave antenna design are also highlighted.

Various future directions and opportunities are highlighted in this review. The design challenges of mmWave array antennas are presented along with the need for robust RF frontend circuity. The challenges of on-chip antennas and integrated system design are highlighted to devise solutions for them. Novel research aspects of efficient MIMO and beamforming techniques associated with mmWave communication are explored. Moreover, the potential of THz communication and THz antennas, along with their challenges, is presented for the next generation of wireless communication. Moreover, light is shed on the capabilities of distributed antenna systems to provide seamless connectivity at mmWave bands. The role of machine learning to design smart antennas is emphasized, which is a demanding area of research. Furthermore, the irrefutable potential of IRS as a key enabler for the next generation of wireless communication is looming. In this view, we accentuated the promising potential of IRS-assisted smart factories to ensure URLLC in Industry 4.0 and beyond.

As a final remark, the 60 GHz mmWave communication with smart antennas holds a promising potential for future industrial automation systems. The use of the huge bandwidth of the mmWave spectrum can pave the way for a wide range of capabilities for Industry 4.0 and beyond. For instance, smart robots, cognitive machines, and other industrial automation systems enable them to actively interact with objects and safely navigate their surroundings using wireless cameras and vision technology. This will facilitate them to become accustomed to changing manufacturing-line conditions, thus unfolding a whole new world of industrial automation possibilities in Industry 4.0 and beyond.

## Figures and Tables

**Table 1:** Comparison of the related contributions of selected literature at 60 GHz band.

Ref.	Comments
[1]	Discussion on the differences between Industry 4.0 and Industry 5.0, the co-existence of these two, and some enabling technologies.
[4]	Review of IoT, big data, and cloud computing for Industry 4.0-based healthcare
[7]	Discussion on cyber-physical systems for Industrial IoT in Industry 4.0.
[24]	A survey of potential applications of Industry 5.0 such as intelligent healthcare, cloud manufacturing, supply chain management, and manufacturing production
[45]	Discussed opportunities and challenges of 60 GHz mmWave communication for industrial environment.
[46]	Highlighted the potential of 60 GHz communication for factory automation scenarios.
[51]	Overview of IEEE 802.11ay standard, as well as new PHY and MAC specifications based on IEEE 802.11ad, MIMO enhanced channel access and beamforming training.
[52]	Design concerns for the IEEE 802.11ad standard, as well as solutions for overcoming mmWave communication problems.
[53]	Industrial perspective of using 60 GHz WiGig communication.
[58]	Review of beamforming training, design issues, channel bonding and aggregation, channel access, and channel allocation in IEEE 802.11ay.
[59]	A detailed survey of 60 GHz radio transceivers, antennas, low-noise amplifiers, power amplifiers, mixers, etc.
[65]	Review of various mmWave antenna designs from 10 to 100 GHz.
[66]	Discussion on 60 GHz radio, link budget, channel propagation, RF front end architecture, and antenna solutions.
**This work**	Review of URLLC requirements in Industry 4.0 and beyond, overview of potential of 60 GHz mmWave band for industrial communication, analysis of wireless standards and protocols at 60 GHz band. Review of various 60 GHz mmWave antennas for Industry 4.0 and beyond and their design challenges. Inclusive discussion on the prospects and research opportunities of 60 GHz mmWave communication and PHY-based solutions for Industry 4.0 and beyond.

**Table 2:** The 60 GHz mmWave ISM band standards suitable for indoor industrial communication.

IEEE Standard	Forum Type	Peak Data Rate (Gbps)	Bandwidth (GHz)
IEEE 802.11ay	International standard	100	8.64
IEEE 802.11ad	Industry consortium	8	2.16
IEEE 802.15.3c	International standard	5.7	<3
WirelessHD	Industry consortium	4	2
ECMA387	International standard	4.032	2.16

**Table 3:** Categories of 60 GHz mmWave antennas with benefits and drawbacks.

Antenna Type	Advantages	Disadvantages
Microstrip and PCB antennas	Compact, low cost, easy fabrication, light weight, easily integrable with other RF circuitry	High substrate loss, conductor and dielectric loss, feed radiation issues, impedance matching issues, bandwidth issues for thick substrates
On-chip integrated antennas	Compact, low power, light weight, low profile and multifunctional	Low gain, low efficiency, high radiation losses, complex fabrication, and complex design rules
Leaky wave and surface wave antennas	Low fabrication cost, planar tunability, no requirement of phase shifters usually	Low efficiency usually due to traveling wave, scanning angle varies with frequency, complex design considerations

**Table 4:** Summary of various 60 GHz antenna designs available in the literature.

Ref.	Antenna Technology	Antenna Type	Array Configuration	Peak Gain (dBi)	Size (mm × mm)
[90]	PCB	Integrated horn	Single unit structure	14.6	-
[85]	PCB	Monopole array	1 × 2	11.6	20.64 × 20
[96]	PCB	SIW coplanar fed slot	Linear array	12	30 × 5
[82]	PCB	T-slot planar	Single element	8.77	11.7 × 9.8
[84]	PCB	CP substrate-integrated cavity	4 × 4	20	30 × 30
[97]	PCB	Microstrip CP array	2 × 2	16	20 × 20
[120]	PCB	Dielectric resonator	2 × 2	11.43	-
[132]	PCB	SIW-based leaky wave	Linear array	14.5	23 × 3
[127]	PCB	SIW fractal antenna	Single SIW	4.57	4.1 × 8.6
[130]	PCB	SIW fractal antenna	Single SIW	7.9	6.5 × 9.6
[110]	LTCC	CP SIW	4 × 4	18.2	18.6 × 18.6
[107]	LTCC	Parasitic microstrip patches	4 × 4	10.5	10.1 × 8.5
[104]	LTCC	Planar aperture	16 × 16	24.6	37 × 37
[111]	LTCC	Patch with SIW feed	4 × 4	16.7	≈20 × 20
[105]	LTCC	Patch	4 × 4	17.1	13 × 13
[114]	LTCC	U-slot patch	4 × 4	16	14 × 16
[115]	LTCC	Helical	4 × 4	14	12 × 10
[103]	On-chip	Yagi	-	-8	Chip size 1.1 × 0.95
[98]	On-chip	MEMS based	9 × 9	23.3	24.75 × 24.75
[99]	On-chip	Folded slot silicon integrated	-	3.9	-

## Data Availability

Not applicable.

## Abbreviations

The following abbreviations are used in this paper.

3GPP	Third-Generation Partnership Project
5G	Fifth Generation
6G	Sixth Generation
CMOS	Complementary Metal Oxide Semiconductor
FPGA	Field-Programmable Gate Array
IC	Integrated Circuit
IoT	Internet of Things
ISM	Industrial Scientific and Medical
M2M	Machine to Machine
mGbps	Multi-Gigabits per Second
MIMO	Multiple-Input-Multiple-Output
ML	Machine Learning
mmWave	Millimeter Wave
NR	New Radio
P2P	Point-to-Point
P2MP	Point-to-Multipoint
PIN	Positive Intrinsic Negative
SNR	Signal-to-Noise Ratio
SiP	System-in-Package
SoC	System-on-Chip
URLLC	Ultra-Reliable and Low-Latency Communication
WiGig	Wireless Gigabit
